# Photosynthesis-Inhibiting Activity of *N*-(Disubstituted-phenyl)-3-hydroxynaphthalene-2-carboxamides †

**DOI:** 10.3390/molecules26144336

**Published:** 2021-07-17

**Authors:** Jiri Kos, Tomas Gonec, Michal Oravec, Izabela Jendrzejewska, Josef Jampilek

**Affiliations:** 1 Department of Biochemistry, Faculty of Medicine, Masaryk University, Kamenice 5, 62500 Brno, Czech Republic; 2 Department of Chemical Drugs, Faculty of Pharmacy, Masaryk University, Palackeho tr. 1946/1, 61200 Brno, Czech Republic; t.gonec@seznam.cz; 3 Global Change Research Institute of the Czech Academy of Sciences, Belidla 986/4a, 60300 Brno, Czech Republic; oravec.m@czechglobe.cz; 4 Institute of Chemistry, University of Silesia, Szkolna 9, 40007 Katowice, Poland; izabela.jendrzejewska@us.edu.pl; 5 Department of Analytical Chemistry, Faculty of Natural Sciences, Comenius University, Ilkovicova 6, 84215 Bratislava, Slovakia; josef.jampilek@gmail.com

**Keywords:** hydroxynaphthalene-carboxamides, PET inhibition, spinach chloroplasts, structure-activity relationships

## Abstract

A set of twenty-four 3-hydroxynaphthalene-2-carboxanilides, disubstituted on the anilide ring by combinations of methoxy/methyl/fluoro/chloro/bromo and ditrifluoromethyl groups at different positions, was prepared. The compounds were tested for their ability to inhibit photosynthetic electron transport (PET) in spinach (*Spinacia oleracea* L.) chloroplasts. *N*-(3,5-Difluorophenyl)-, *N*-(3,5-dimethylphenyl)-, *N*-(2,5-difluorophenyl)- and *N*-(2,5-dimethylphenyl)-3-hydroxynaphthalene-2-carboxamides showed the highest PET-inhibiting activity (IC_50_ ~ 10 µM) within the series. These compounds were able to inhibit PET in photosystem II. It has been found that PET-inhibiting activity strongly depends on the position of the individual substituents on the anilide ring and on the lipophilicity of the compounds. The electron-withdrawing properties of the substituents contribute towards the PET activity of these compounds.

## 1. Introduction

Due to population growth, there is a constant pressure on farmers to multiply yields to ensure sufficient food. On the other hand, this challenge is difficult to meet due to deteriorating conditions for agriculture, such as the loss of quality agricultural land, desiccation or, conversely, heavy rains and floods, climate change and the rise of many plant and crop destroyers. One way to combat pathogens of plants is to use pesticides to help farmers increase productivity per hectare by protecting plants from pests, diseases and weeds. For example, food crops must compete with approximately 30,000 weed species. Herbicides are still used widely around the world because manual weeding has never been an effective method of weed control, especially when large-scale farming is used. Herbicides are often used instead of tillage because the use of herbicides reduces erosion, fuel consumption, greenhouse gas emissions and nutrient leakage, and saves water compared to plowing. Of course, the question remains as to what extent the negative chemical effects of herbicides harm non-target organisms and degrade soil and water resources [1,2,3].

Herbicides can be classified according to the type/chemical structure of the active ingredient, mechanism of action, method and time of application, mobility, type of formulation or residual effect [4,5,6]. There are currently about 20 different mechanisms of action of herbicides [4,5,6,7,8,9,10,11], but over 50% of commercially available herbicides act by reversibly binding to photosystem II (PS II), resulting in disruption of photosynthetic electron transport (PET) [4,6,7,8]. PS II uses light energy to oxidize water and reduce plastoquinone, which consists of parts Q_A_ and Q_B_. The plastoquinone Q_A_ acting as a single electron acceptor is permanently bound to PS II; the plastoquinone Q_B_ acting as a two-electron acceptor is loosely bound; after reduction, it separates from the reaction center and diffuses into the hydrophobic membrane nucleus, the Q_B_ binding site being occupied by the oxidized molecule plastoquinone [12]. Herbicides belonging to inhibitors of PS II inhibit photosynthetic electron transfer (PET) by binding to the Q_B_ binding niche on the D_1_ protein of the PS II complex in chloroplast thylakoid membranes, leading to inhibition of PET from Q_A_ to Q_B_, blocking CO_2_ fixation and inhibition of ATP production [6,9,10,11].

Studies of large libraries of structurally diverse PS II inhibitors have confirmed the hydrophobic nature of the binding domain, with lipophilicity being the dominant determinant of Hill inhibitory activity [12,13,14,15,16,17,18]. Significant amounts of herbicides acting as PET inhibitors in PS II contain an amide (–CONH–) and/or carbamate (–HNCOO–) bond in their structure that is capable of forming hydrogen bonds between the amide/carbamate group and target proteins in the photosynthetic centers of thylakoid membranes, leading to conformational changes and PET inhibition [19,20,21,22,23,24,25]. Both the *N*- and *O*-terminal ends of the CONH linker are substituted and the substituents further modify the bond properties and strength of the basic scaffold [26]. Amides are thought to be inhibitors of PS II, causing the displacement of Q_B_ from its binding pocket in the D_1_ protein [27], and halogenated substituents have been found to contribute to increased PET inhibitory activity [18,23,24,28,29,30].

Our team has long been investigating the effects of a wide range of variously substituted napthalenecarboxanilides [23,24,27,29,30,31] and quinolinecarboxanilides [32] on PS II. A series of ring-monosubstituted anilides of 3-hydroxynaphthalene-2-carboxylic acid was published by Kos et al. [29] and some interesting biological activity was found, including herbicidal activity. Since monosubstituted derivatives of 3-hydroxy-*N*-arylnaphthalene-2-carboxanilides showed PET inhibition in spinach chloroplasts (*Spinacia oleracea* L.), select new, variously disubstituted, derivatives were evaluated for their PET-inhibiting activity.

## 2. Results and Discussion

### 2.1. Chemistry

**Scheme 1:**
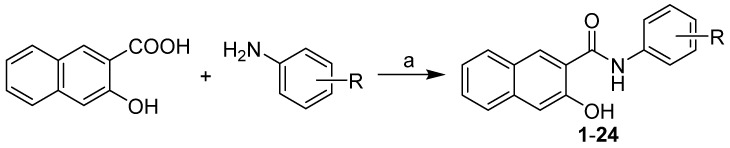
Synthesis of 3-hydroxy-*N*-arylnaphthalene-2-carboxanilides **1**–**24**. Reagents and conditions: (**a**) PCl_3_, chlorobenzene, MW, 45 min [29,31].

All compounds were prepared by the reaction of 3-hydroxynaphthalene-2-carboxylic acid with appropriate disubstituted anilines with the addition of phosphorus trichloride in dry chlorobenzene under microwave conditions (Scheme 1) [29,31], which resulted in a series of target *N*-(disubstituted-phenyl)-3-hydroxynaphthalene-2-carboxamides **1**–**24**, see Table 1.

**Figure 1:**
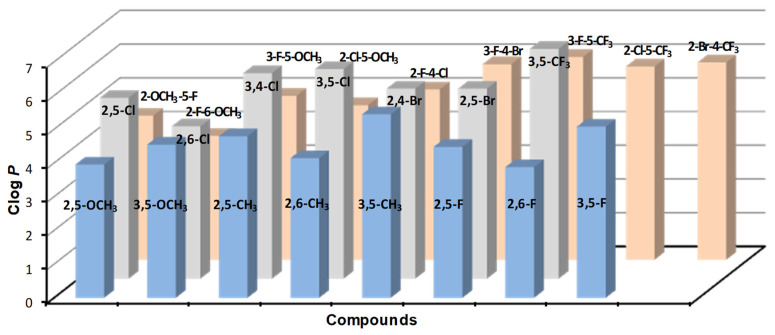
Graphical comparison of lipophilicity of investigated compounds expressed as Clog *P,* predicted by ChemBioDraw Ultra 13.0.

Lipophilicity is an extremely important parameter in the design of any biologically active compound, as it primarily ensures sufficient penetration across biological membranes to reach the target site of action [33]. In general, it can be stated that a higher value of lipophilicity is required for agrochemicals acting in plant leaves due to the permeability of a stronger and more lipophilic cuticle [34]. Lipophilicity, expressed as Clog *P* values (predicted by ChemBioDraw Ultra 13.0), of the investigated compounds is listed in Table 1. Clog *P* values ranged from 3.6 (compound **17**, R = 2-F-6-OCH_3_) to 6.8 (compound **15**, R = 3,5-CF_3_). When comparing the general effect of substituents at the same positions on lipophilicity, the order of the groups with respect to the increasing contribution of lipophilicity is as follows: OCH_3_ < F < CH_3_ < Cl < Br < CF_3_; thus, in general, any substitution by a fluorine or methoxy moiety significantly decreases lipophilicity. In addition to the type of substituent, their mutual position on the aniline ring also has a significant effect on the lipophilicity value. For example, in series with dichlorinated derivatives, lipophilicity increases as follows: 2.6 < 2.5 < 3.4 < 3.5. In series with different moieties of disubstituted compounds, the Clog *P* values are significantly decreased when a fluorine or methoxy moiety is introduced, especially in positions C_(2)_′ and C_(6)_′, as mentioned above. The predicted Clog *P* values are presented in the illustrated order in Figure 1, where they are simultaneously divided into three groups according to the nature of the substitution. The first group consists of methoxy-, methyl- and fluoro-disubstituted compounds **1**–**8**; the second group consists of dichloro, dibromo and 3,5-CF_3_ derivatives **9**–**15**; and derivatives **16**–**24,** disubstituted by two different substituents, are in the third group. This division proves important for the description of PET inhibition, see below.

Electronic contributions of substituents are another important parameter, especially for substituted aromatic rings (anilines, phenols). The electron σ parameters of the whole substituted anilide ring, predicted by the ADC/Percepta program, are listed in Table 1. As with the lipophilicity values, the σ values are in a wide range. Based on the results of the prediction program, the weakest electron-withdrawing properties have the substitution 2,5-OCH_3_-Ph of compound **1** (σ = 0.08), while the strongest electron-withdrawing properties have fluoro-substituted derivative **7** (2,6-F-Ph, σ = 1.44). These values affect the electron density at the amide linker and thus the overall binding to the putative site of action of these compounds, which is on the acceptor side of PS II, at the section between P_680_ (primary donor of PS II) and Q_B_ [23,24,25,29].

### 2.2. Inhibition of Photosynthetic Electron Transport (PET) in Spinach Chloroplasts

The PET-inhibition of the studied compounds was expressed by the negative logarithm of the IC_50_ value (concentration (in µM) of the compounds causing a 50% decrease in the oxygen evolution rate relative to the untreated control). The evaluated disubstituted 3-hydroxynaphthalene-2-carboxanilides showed a wide range of PET inhibition in spinach (*Spinacia oleracea* L.) chloroplasts with the IC_50_ values ranging from 9.8 to 1405 µM, see Table 1. *N*-(3,5-Difluorophenyl)-(**8**) and *N*-(3,5-dimethylphenyl)-(**5**), *N*-(2,5-difluorophenyl)-(**6**) and *N*-(2,5-dimethylphenyl)-(**3**) 3-hydroxynaphthalene-2-carboxamides demonstrated the highest PET-inhibiting activity (IC_50_ ~ 10 µM) within the whole investigated series. Acceptable activity was also found for *N*-(2-chloro-5-trifluoromethylphenyl)-(**23**) and *N*-(3,5-ditrifluoromethylphenyl)-3-hydroxynaphthalene-2-carboxamides (**15**) with IC_50_ 13.2 and 15.9 µM, respectively. On the other hand, derivatives **21** (3-F-4-Br), **24** (2-Br-4-CF_3_) and **20** (R = 2-F-4-Cl) were completely inactive (IC_50_ = 527, 621 and 1405 µM, respectively).

**Figure 2:**
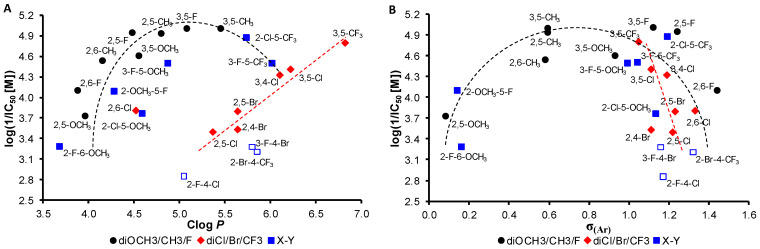
Dependence of PET-inhibiting activity log(1/IC_50_ [M]) of all discussed compounds **1**–**24** in spinach chloroplasts on lipophilicity expressed as Clog *P* (**A**) and electronic σ parameters of whole *N*-aryl part of individual anilides (**B**). Empty squares are not involved in SAR discussion due to their inactivity.

The results of this screening indicate that the position of the substituents is crucial for the activity, with the 3,5 positions being the most preferred (i.e., both *meta* positions are substituted). However, 2,5-disubstituted derivatives also showed PET-inhibiting activity when substituted with moieties with suitable properties, including electronic properties and lipophilicity. As mentioned above, lipophilicity tends to affect biological activity. The dependence of the PET-inhibiting activity, expressed as log(1/IC_50_ [M]), of the investigated compounds in spinach chloroplasts on lipophilicity (Clog *P*) is shown in Figure 2A. It can be stated that most of the evaluated compounds substituted by OCH_3_/CH_3_/F can be traced to a quasi-parabolic dependence with the optimum Clog *P* ca. 5. The active compounds have a range of lipophilicity values from 4.4 to 5.7. On the other hand, a linear dependence can be observed for the dichloro-, dibromo- and bis(trifluoromethyl)-substituted compounds, i.e., markedly lipophilic groups. The inhibition of PET increases with increasing lipophilicity. 

Figure 2B shows the dependence of the PET-inhibiting activity, expressed as log(1/IC_50_ [M]), on the electronic σ_(Ar)_ properties of the whole anilide substituents. As can be seen, electronic properties play a secondary role compared to lipophilicity and substituent position; however, the quasi-parabolic (for OCH_3_/CH_3_/F substituted compounds) or linear (for disubstituted compounds by Cl/Br/CF_3_ moieties) trend is evident. It can be stated that electron-withdrawing properties in the range of σ_(Ar)_ from approximately 0.6 to 1.2 are preferred.

Based on the structural similarity of the test compounds to previously performed experiments with salicylanilides or hydroxynaphthanilides, the same mechanism of action can be supposed, i.e., inhibition on the acceptor side of PS II, at the section between P_680_ (primary donor of PS II) and plastoquinone Q_B_ [20,21,22,23,24,27,29,30,31]. Furthermore, it should be noted that plastoquinone Q_B_ on the acceptor side of PS II has been found to be the site of inhibitory action of other amide-based derivatives [6,13,14,15,25,35], such as *N*-phenylpyrazine-2-carboxamides [19], *N*-substituted 2-aminobenzothiazoles [22] or 8-hydroxyquinoline-2-carboxanilides [32].

## 3. Materials and Methods

### 3.1. General Information

All reagents were purchased from Merck (Sigma-Aldrich, St. Louis, MO, USA) and Alfa (Alfa-Aesar, Ward Hill, MA, USA). Reactions were performed using a CEM Discover SP microwave reactor (CEM, Matthews, NC, USA). The melting points were determined on a Kofler hot-plate apparatus HMK (Franz Kustner Nacht KG, Dresden, Germany) and are uncorrected. Infrared (IR) spectra were recorded on a Smart MIRacle™ ATR ZnSe for Nicolet™ Impact 410 Fourier-transform IR spectrometer (Thermo Scientific, West Palm Beach, FL, USA). The spectra were obtained by the accumulation of 256 scans with 2 cm^−1^ resolution in the region of 4000–650 cm^−1^. All ^1^H- and ^13^C-NMR spectra were recorded in dimethyl sulfoxide-*d*_6_ (DMSO-*d*_6_) at 600 MHz for ^1^H and 150 MHz for ^13^C, on an Agilent VNMRS 600 MHz system (Agilent Technologies, Santa Clara, CA, USA). The ^1^H and ^13^C chemical shifts (δ) are reported in ppm. High-resolution mass spectra were measured using a high-performance liquid chromatograph Dionex UltiMate^®^ 3000 (Thermo Scientific, West Palm Beach, FL, USA) coupled with an LTQ Orbitrap XL^TM^ Hybrid Ion Trap-Orbitrap Fourier Transform Mass Spectrometer (Thermo Scientific) equipped with a HESI II (heated electrospray ionization) source in positive and negative mode.

### 3.2. Synthesis

#### General Procedure for the Synthesis of *N*-(Disubstituted phenyl)-3-hydroxynaphthalene-2-carboxamides **1**–**24**

3-Hydroxynaphthalene-2-carboxylic acid (0.5 g, 2.65 mM) was suspended in dry chlorobenzene (20 mL) at ambient temperature and phosphorus trichloride (0.12 mL, 1.35 mM), and the corresponding substituted aniline (2.65 mM) was added dropwise. The reaction mixture was transferred to the microwave reactor, where the synthesis was performed (1st phase: 10 min, 100 °C; 2nd phase: 15 min, 120 °C; 3rd phase: 20 min, 130 °C; max 500 W). The mixture was then cooled to 60 °C, and the solvent was removed under reduced pressure. The residue was washed sequentially with hydrochloric acid and water, and the crude product was recrystallized from EtOH. All the compounds are presented in Table 1.

The synthesis and analytical data for anilides **1–12, 15** and **21–24** were described previously [31].

*N-(2,4-Dibromophenyl)-3-hydroxynaphthalene-2-carboxamide* (**13**). Yield 56%; mp 241–243 °C; IR (cm^−1^): 3221; 1641; 1625; 1603; 1575; 1524; 1462; 1448; 1398; 1363; 1345; 1321; 1290; 1240; 1206; 1175; 1146; 1081; 1035; 951; 913; 878; 867; 846; 825; 791; 767; 737; 688; ^1^H NMR (DMSO-*d*_6_), δ: 11.97 (s, 1H), 11.07 (s, 1H), 8.70 (s, 1H), 8.42 (d, *J* = 8.8 Hz, 1H), 7.99 (d, *J* = 8.2 Hz, 1H), 7.97 (d, J = 2.2 Hz, 1H), 7.78 (d, *J* = 8.3 Hz, 1H), 7.66 (dd, *J* = 2.2 Hz, *J* = 8.8 Hz, 1H), 7.53 (ddd, *J* = 1.2 Hz, *J* = 6.8 Hz, *J* = 8.3 Hz, 1H), 7.38 (s, 1H), 7.37 (ddd, *J* = 1.1 Hz, *J* = 6.8 Hz, *J* = 8.2 Hz, 1H); ^13^C NMR (DMSO-*d*_6_), δ: 163.6, 152.6, 136.2, 136.1, 134.4, 132.8, 131.3, 129.1, 128.6, 127.2, 125.7, 124.4, 124.0, 120.4, 116.3, 114.9, 110.8; HR-MS C_17_H_12_O_2_NBr_2_ [M + H]^+^ calculated 419.9229 *m/z*, found 419.9237 *m/z*.

*N-(2,5-Dibromophenyl)-3-hydroxynaphthalene-2-carboxamide* (**14**). Yield 49%; mp 233–235 °C; IR (cm^−1^): 3190; 1636; 1622; 1597; 1568; 1506; 1447; 1393; 1360; 1344; 1250; 1192; 1174; 1147; 1080; 1069; 1029; 962; 915; 902; 868; 848; 796; 770; 750; 736; ^1^H NMR (DMSO-*d*_6_), δ: 12.02 (s, 1H), 11.14 (s, 1H), 8.74 (d, *J* = 2.3 Hz, 1H), 8.71 (s, 1H), 8.00 (d, *J* = 8.2 Hz, 1H), 7.79 (d, *J* = 8.3 Hz, 1H), 7.69 (d, *J* = 8.5 Hz, 1H), 7.54 (ddd, *J* = 1.2 Hz, *J* = 6.8 Hz, *J* = 8.3 Hz, 1H), 7.39 (s, 1H), 7.38 (ddd, *J* = 1.1 Hz, *J* = 6.8 Hz, *J* = 8.2 Hz, 1H), 7.32 (dd, *J* = 2.4 Hz, *J* = 8.5 Hz, 1H); ^13^C NMR (DMSO-*d*_6_), δ: 163.6, 152.5, 138.1, 136.2, 134.2, 133.0, 129.1, 128.7, 128.1, 127.2, 125.7, 125.0, 124.0, 120.9, 120.3, 112.7, 110.8; MS C_17_H_12_O_2_NBr_2_ [M + H]^+^ calculated 419.9229 *m/z*, found 419.9239 *m/z*.

*N-(5-Fluoro-2-methoxyphenyl)-3-hydroxynaphthalene-2-carboxamide* (**16**). Yield 80%; mp 198–203 °C; IR (cm^−1^): 3194, 1640, 1625, 1615, 1601, 1538, 1488, 1432, 1393, 1356, 1346, 1249, 1214, 1176, 1148, 1065, 1038, 975, 866, 838, 786, 731, 711; ^1^H-NMR (DMSO-*d*_6_), δ: 11.86 (s, 1H), 11.25 (s, 1H), 8.70 (s, 1H), 8.40 (dd, *J* = 11.0 Hz, J=3.3 Hz, 1H), 7.93 (d, *J* = 8.1 Hz, 1H), 7.78 (d, *J* = 8.4 Hz, 1H), 7.53 (t, *J* = 7.5 Hz, 1H), 7.37 (s, 1H), 7.36 (t, *J* = 7.5 Hz, 1H), 7.12 (dd, *J* = 9.2 Hz, J = 5.1 Hz, 1H), 6.92 (td, *J* = 8.6 Hz, *J* = 3.3 Hz, 1H), 3.92 (s, 3H); ^13^C-NMR (DMSO-*d*_6_), δ: 163.0, 156.0 (d, *J* = 232.2 Hz), 152.4, 144.8 (d, *J* = 1.8 Hz), 136.0, 132.9, 129.1, 129.0 (d, *J* = 12.9 Hz), 128.5, 127.2, 125.7, 123.9, 121.0, 111.7 (d, *J* = 9.1 Hz), 110.8, 109.0 (d, *J* = 22.8 Hz), 106.9 (d, *J* = 29.6 Hz), 57.0; HR-MS: C_18_H_13_FNO_3_ [M − H]^−^ calculated 310.0885 *m/z*, found 310.0881 *m/z*.

*N-(2-Fluoro-6-methoxyphenyl)-3-hydroxynaphthalene-2-carboxamide* (**17**). Yield 66%; mp 138–144 °C; IR (cm^−1^): 3259, 2836, 1651, 1622, 1596, 1532, 1515, 1506, 1466, 1438, 1279, 1249, 1216, 1167, 1146, 1087, 900, 873, 834, 789, 767, 747, 728; ^1^H-NMR (DMSO-*d*_6_), δ: 11.76 (s, 1H), 10.22 (s, 1H), 8.69 (s, 1H), 7.93 (d, *J* = 8.8 Hz, 1H), 7.78 (d, *J* = 8.4 Hz, 1H), 7.54 (t, *J* = 7.3 Hz, 1H), 7.30–7.40 (m, 3H), 6.99 (d, *J* = 8.4 Hz, 1H), 6.94 (t, *J* = 9.0 Hz, 1H), 3.84 (s, 3H); ^13^C-NMR (DMSO-*d*_6_), δ: 166.3, 158.1 (d, *J* = 246.4 Hz), 155.8 (d, *J* = 5.3 Hz), 154.6, 136.2, 131.0, 128.9, 128.5, 128.2 (d, *J* = 10.7 Hz), 126.8, 125.8, 123.9, 118.7, 113.7 (d, *J* = 15.3 Hz), 110.9, 107.9 (d, *J* = 26.4 Hz), 107.7, 56.3; HR-MS: C_18_H_13_FNO_3_ [M − H]^−^ calculated 310.0885 *m/z*, found 310.0880 *m/z*.

*N-(3-Fluoro-5-methoxyphenyl)-3-hydroxynaphthalene-2-carboxamide* (**18**). Yield 59%; mp 227–230 °C; IR (cm^−1^): 3147, 1644, 1622, 1595, 1557, 1520, 1456, 1448, 1359, 1261, 1224, 1212, 1191, 1141, 1129, 1063, 999, 987, 872, 858, 816, 767, 745, 690; ^1^H-NMR (DMSO-*d*_6_), δ: 11.12 (s, 1H), 10.64 (s, 1H), 8.41 (s, 1H), 7.93 (d, *J* = 8.4 Hz, 1H), 7.76 (d, *J* = 8.1 Hz, 1H), 7.51 (t, *J* = 7.0 Hz, 1H), 7.32-7.40 (m, 3H), 7.21 (s, 1H), 6.62 (d, *J* = 11.0 Hz, 1H), 3.78 (s, 3H); ^13^C-NMR (DMSO-*d*_6_), δ: 165.7, 162.9 (d, *J* = 238.5 Hz), 160.7 (d, *J* = 12.9 Hz), 153.3, 140.6 (d, *J* = 13.7 Hz), 135.7, 130.5, 128.7, 128.1, 126.9, 125.8, 123.8, 122.5, 110.5, 102.0 (d, *J* = 2.0 Hz), 99.4 (d, *J* = 27.3 Hz), 97.0 (d, *J* = 25.0 Hz), 55.6; HR-MS: C_18_H_13_FNO_3_ [M − H]^−^ calculated 310.0885 *m/z*, found 310.0881 *m/z*.

*N-(2-Chloro-5-methoxyphenyl)-3-hydroxynaphthalene-2-carboxamide* (**19**). Yield 58%; mp 187–188 °C; IR (cm^−1^): 3177, 2954, 2834, 1638, 1624, 1598, 1539, 1462, 1447, 1427, 1358, 1305, 1274, 1262, 1220, 1167, 1147, 1135, 1063, 1028, 960, 916, 866, 845, 787, 771, 745, 719; ^1^H-NMR (DMSO-*d_6_*), δ: 11.97 (s, 1H), 11.17 (s, 1H), 8.73 (s, 1H), 8.25 (d, *J =* 2.9 Hz, 1H), 7.99 (d, *J =* 8.2 Hz, 1H), 7.78 (d, *J =* 8.3 Hz, 1H), 7.53 (ddd, *J =* 8.3 Hz, *J =* 6.8 Hz, *J =* 1.2 Hz, 1H), 7.46 (d, *J =* 8.8 Hz, 1H), 7.38 (ddd, *J =* 8.2 Hz, *J =* 6.8 Hz, *J =* 1.2 Hz, 1H), 7.38 (s, 1H), 6.78 (dd, *J =* 8.8 Hz, *J =* 3.0 Hz, 1H), 3.80 (s, 3H); ^13^C-NMR (DMSO-*d_6_*), δ: 163.4, 158.5, 152.5, 136.1, 132.9, 129.6, 129.1, 128.6, 127.2, 125.7, 124.0, 120.6, 114.2, 110.8, 110.4, 108.0, 55.5; HR-MS: C_18_H_15_ClNO_3_ [M + H]^+^ calculated 328.0735 *m*/*z*, found 328.0737 *m*/*z.*

*N-(4-Chloro-2-fluorophenyl)-3-hydroxynaphthalene-2-carboxamide* (**20**). Yield 75%; mp 267–269 °C; IR (cm^−1^): 3194, 1647, 1627, 1601, 1552, 1489, 1449, 1414, 1393, 1357, 1338, 1259, 1207, 1174, 1147, 1118, 1064, 951, 918, 897, 870, 841, 820, 767, 740, 722, 667; ^1^H NMR (DMSO-*d*_6_) δ: 11.85 (s, 1H), 10.97 (s, 1H), 8.66 (s, 1H), 8.37 (t, *J* = 8.7 Hz, 1H), 7.97 (d, *J* = 8.2 Hz, 1H), 7.77 (d, *J* = 8.3 Hz, 1H), 7.56 (dd, *J* = 2.4 Hz, *J* = 10.8 Hz, 1H), 7.52 (ddd, *J* = 1.2 Hz, *J* = 6.8 Hz, *J* = 8.3 Hz, 1H), 7.37 (ddd, *J* = 1.1 Hz, *J* = 6.8 Hz, *J* = 8.2 Hz, 1H), 7.36 (s, 1H), 7.34 (ddd, *J* = 1.2 Hz, *J* = 2.4 Hz, *J* = 8.8 Hz, 1H). ^13^C NMR (DMSO-*d*_6_), δ: 163.8, 152.8 (d, *J* = 247.5 Hz), 152.8, 136.1, 132.4, 129.0, 128.5, 127.9 (d, *J* = 10.0 Hz), 127.1, 125.7, 125.7 (d, *J* = 10.7 Hz), 124.9 (d, *J* = 3.4 Hz), 124.0, 123.6, 120.4, 115.9 (d, *J* = 23.1 Hz), 110.9; HR-MS: C_17_H_12_ClFNO_2_ [M + H]^+^ calculated 316.0535 *m/z*, found 316.0535 *m/z*.

### 3.3. Study of Inhibition of Photosynthetic Electron Transport (PET) in Spinach Chloroplasts

Chloroplasts were prepared from spinach (*Spinacia oleracea* L.) according to Kralova et al. [36]. Screening was performed as described previously [e.g., 19–25,31]. A selective herbicide 3-(3,4-dichlorophenyl)-1,1-dimethylurea, DCMU (Diuron^®^, Merck, Darmstadt, Germany) was used as a standard. The results are summarized in Table 1.

## 4. Conclusions

A series of 3-hydroxynaphthalene-2-carboxanilides substituted with two similar or different atoms or groups on the anilide ring was prepared under microwave-assisted conditions and tested for their ability to inhibit photosynthetic electron transport (PET) in spinach (*Spinacia oleracea* L.) chloroplasts. *N*-(3,5-Difluorophenyl)-3-hydroxynaphthalene-2-carboxamide (**8**), *N*-(3,5-dimethylphenyl)-3-hydroxynaphthalene-2-carboxamide (**5**), *N*-(2,5-difluorophenyl)-3-hydroxynaphthalene-2-carboxamide (**6**) and *N*-(2,5-dimethylphenyl)-3-hydroxynaphthalene-2-carboxamide (**3**) exhibited the highest PET-inhibiting activity with their IC_50_ values ranging from 9.8 to 11.6 µM. The C_(3,5)_′ and C_(2,5)_′ disubstituted isomers were found to be the most active among the test compounds. Furthermore, for diOCH_3_/diCH_3_/diF substituted derivatives, a Clog *P* value of approximately 5 is important, while for diCl/diBr/diCF_3_ substituted derivatives, PET inhibition increases with increasing lipophilicity to a Clog *P* value of 6.8 of *N*-(3,5-ditrifluoromethylphenyl)-3-hydroxynaphthalene-2-carboxamides (**15**) with IC_50_ = 15.9 µM. The electronic properties of the substituents play a complementary role and the electron-withdrawing properties (σ_(Ar)_ ca. 0.6 to 1.2) for PET activity seem to be more advantageous. Based on the structural similarity of the investigated compounds with previously published isomers, it can be concluded that these hydroxynaphthanilides inhibit PET in photosystem II.

## Figures and Tables

**Table 1:** Structure of ring-disubstituted 3-hydroxynaphthalene-2-carboxanilides **1**–**24**, calculated values of Clog *P* for compounds, electronic σ parameters of anilide (Ar) and IC_50_ [µM] values related to photosynthetic electron transport (PET) inhibition in spinach chloroplasts of tested compounds in comparison with the 3-(3,4-dichlorophenyl)-1,1-dimethylurea (DCMU) standard.

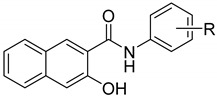				
Comp.	R	Clog *P* ^2^	σ_(Ar)_ ^3^	PET Inhibition IC_50_ [µM]
**1** ^1^	2,5-OCH_3_	3.9563	0.08	183
**2** ^1^	3,5-OCH_3_	4.5463	0.93	24.5
**3** ^1^	2,5-CH_3_	4.7942	0.59	11.6
**4** ^1^	2,6-CH_3_	4.1442	0.58	28.5
**5** ^1^	3,5-CH_3_	5.4442	0.59	9.9
**6** ^1^	2,5-F	4.4799	1.24	11.2
**7** ^1^	2,6-F	3.8799	1.44	78.7
**8** ^1^	3,5-F	5.0799	1.12	9.8
**9** ^1^	2,5-Cl	5.3699	1.22	321
**10** ^1^	2,6-Cl	4.5199	1.33	156
**11** ^1^	3,4-Cl	6.0999	1.19	47.5
**12** ^1^	3,5-Cl	6.2199	1.11	39.2
**13**	2,4-Br	5.6399	1.11	296
**14**	2,5-Br	5.6399	1.23	161
**15** ^1^	3,5-CF_3_	6.8207	1.05	15.9
**16**	2-OCH_3_-5-F	4.2725	0.14	79.1
**17**	2-F-6-OCH_3_	3.6725	0.16	507
**18**	3-F-5-OCH_3_	4.8625	0.99	31.6
**19**	2-Cl-5-OCH_3_	4.5825	1.13	171
**20**	2-F-4-Cl	5.0499	1.17	1405
**21** ^1^	3-F-4-Br	5.7999	1.16	527
**22** ^1^	3-F-5-CF_3_	6.0131	1.04	31.0
**23** ^1^	2-Cl-5-CF_3_	5.7331	1.19	13.2
**24** ^1^	2-Br-4-CF_3_	5.8531	1.32	621
DCMU	–	–	–	2.1

^1^ Compounds described in [31]; ^2^ ChemBioDraw Ultra 13.0 (CambridgeSoft, PerkinElmer Inc., MA, USA); ^3^ calculated using ACD/Percepta ver. 2012 (Advanced Chemistry Development, Toronto, ON, Canada).

## Data Availability

Data is contained within the article.
